# Ten-Year Follow-Up After Chemotherapy and Conversion Surgery for Human Epidermal Growth Factor Receptor 2-Positive Stage IV Esophagogastric Junction Cancer With a Pathological Complete Response: A Case Report

**DOI:** 10.7759/cureus.60178

**Published:** 2024-05-13

**Authors:** Takeharu Enomoto, Shinya Mikami, Takehito Otsubo, Takashi Tsuda, Motohiro Chosokabe

**Affiliations:** 1 Department of Gastroenterological and General Surgery, St. Marianna University School of Medicine, Kawasaki, JPN; 2 Department of Gastrointestinal and General Surgery, St. Marianna University School of Medicine, Kawasaki, JPN; 3 Department of Oncology, Center for Hepato-Biliary-Pancreatic and Digestive Disease, Shonan Fujisawa Tokushukai Hospital, Fujisawa, JPN; 4 Department of Pathology, St. Marianna University School of Medicine, Kawasaki, JPN

**Keywords:** conversion surgery, pathologic complete response, chemotherapy, her2-positive, stage iv gastric cancer

## Abstract

Recent reports have focused on the usefulness of conversion surgery, in which chemotherapy is given to patients with unresectable advanced gastric cancer (GC), and radical surgery is subsequently performed if resection becomes possible; however, no consensus has been reached regarding the usefulness of this strategy. We report on a 74-year-old man who was diagnosed with esophagogastric junction cancer (T3N3M1 (LYM): stage IV). Chemotherapy was chosen and seven courses of S1 + cisplatin (SP) + trastuzumab (HCN) and two courses of S1 + HCN were administered. Approximately 10 months after the start of chemotherapy, the tumor had almost disappeared and we therefore decided to perform conversion surgery. Pathologic examination of the specimen and dissected lymph nodes showed no cancer. Postoperatively, the patient underwent chemotherapy until the second postoperative year, and no metastasis or recurrence was observed for nine years after surgery. Conversion surgery after chemotherapy resulted in recurrence-free survival in this case; however, further studies are needed to elucidate the effect of surgery after chemotherapy for patients with stage IV GC, as chemotherapy continues to evolve.

## Introduction

Gastric cancer (GC) is the leading cause of morbidity and mortality in Japan, and effective treatment strategies are essential. Although recent advances have enabled high response rates in patients with GC, chemotherapy is still recommended for unresectable or recurrent GC. The median survival for patients with stage IV GC is approximately 15 months, and a complete cure is considered difficult to achieve [1]. Some recent studies have reported the usefulness of conversion surgery, in which patients with unresectable advanced GC are treated with chemotherapy, followed by radical surgery if resection becomes possible [2-4]; however, there is currently no consensus on the usefulness of this strategy. In the present case, we performed chemotherapy for esophagogastric junction cancer with left supraclavicular fossa and para-aortic lymph node metastasis, followed by surgery after confirming a complete response. We herein discuss the usefulness of conversion surgery.

This article was previously presented as a meeting abstract at the 2021 Japanese Gastric Cancer Association Annual Meeting on March 3, 2021.

This article was previously posted to the Research Square preprint server on November 21, 2023.

## Case presentation

**Figure 1:**
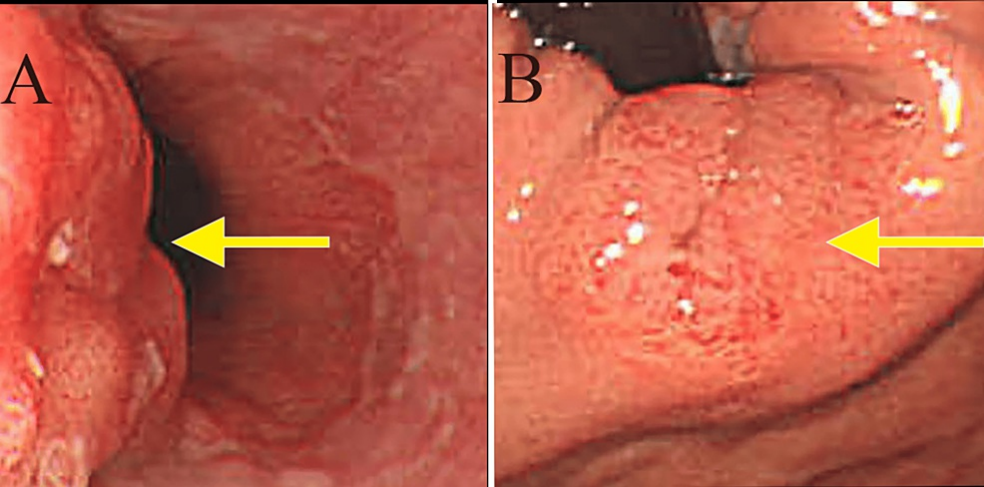
Esophagogastroduodenoscopy (A, B) Images taken before chemotherapy, showing an elevated lesion with ulceration at the esophagogastric junction (40 mm in diameter, type 1 gross type).

**Figure 2:**
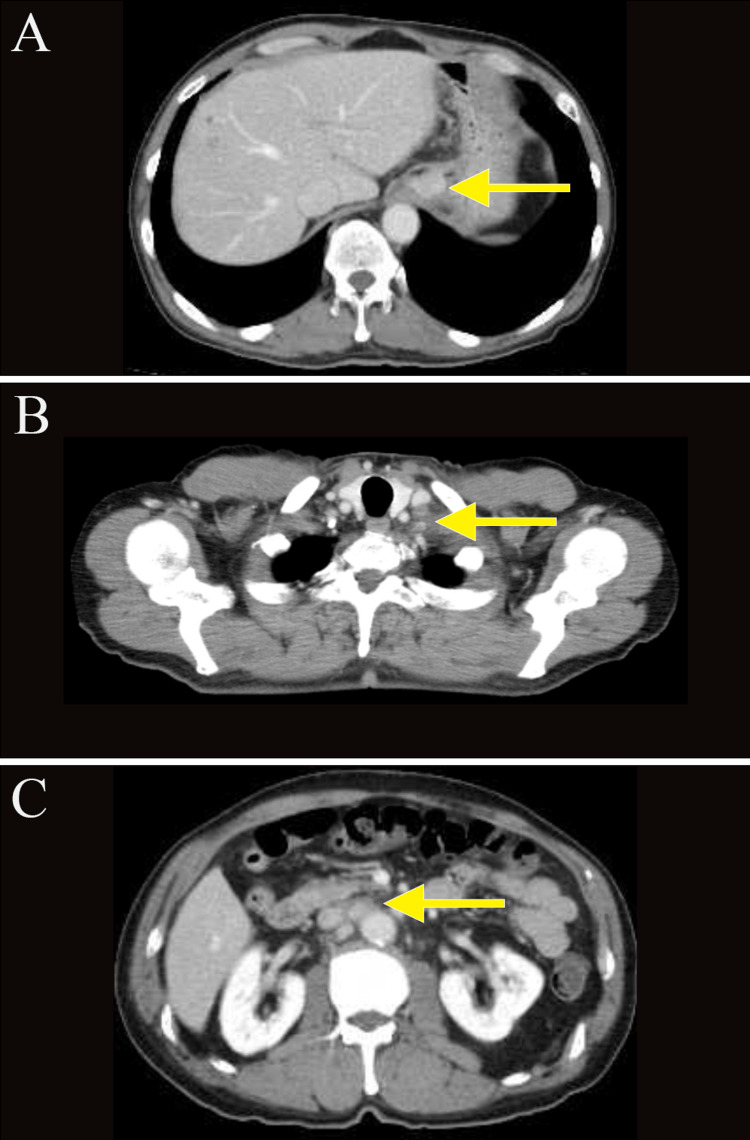
Computed tomography (A-C) Images taken before chemotherapy. Abdominal and cervical computed tomography (CT) scans revealed wall thickening at the hilum and multiple enlarged lymph nodes in the left supraclavicular fossa and around the aorta, from the hilum to the upper abdomen.

**Figure 3:**
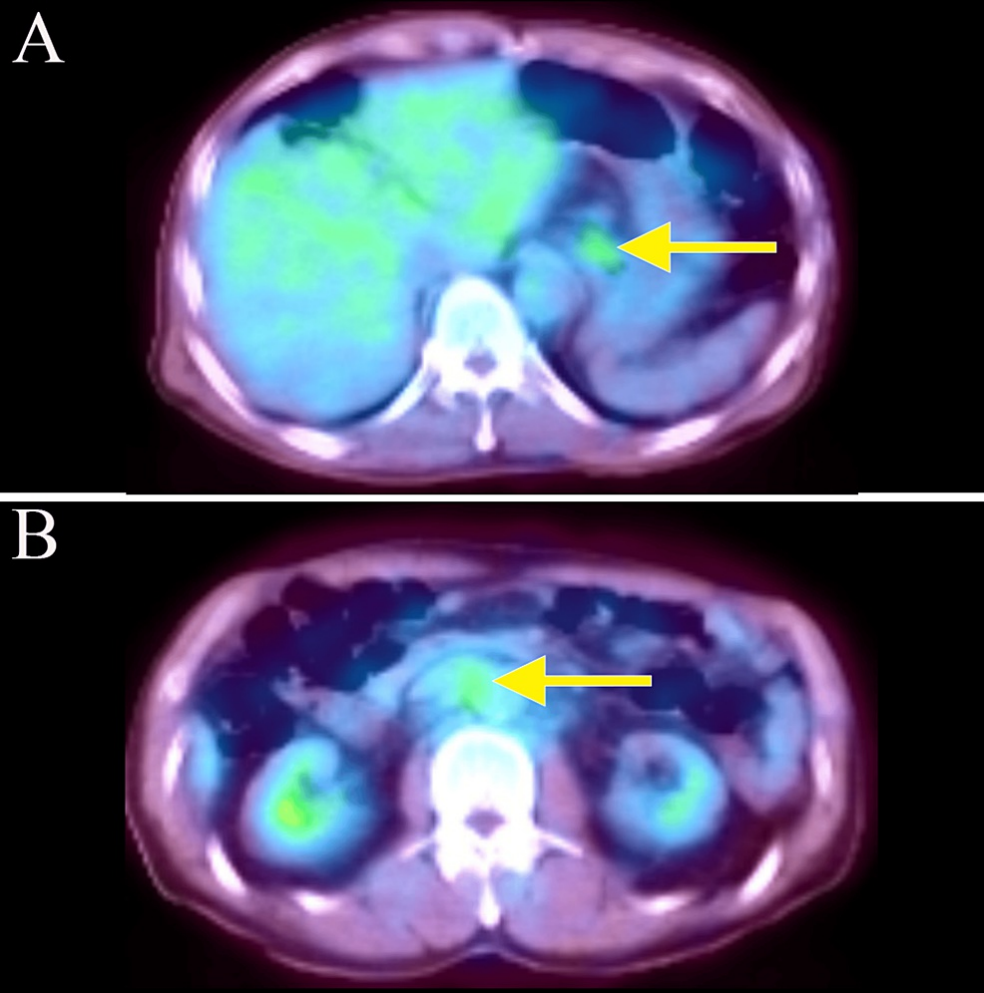
Positron emission tomography-computed tomography (A, B) Image taken before chemotherapy. Positron emission tomography (PET)-CT scans confirmed wall thickening at the hilum and multiple enlarged lymph nodes in the left supraclavicular fossa and around the aorta from the hilum to the upper abdomen. The wall thickening at the hilum had an SUVmax of 3.0; multiple enlarged lymph nodes around the aorta from the hilum to the upper abdomen had SUVmax values ranging from 2.5 to 3.0.

A 74-year-old man presented with a chief complaint of abdominal pain, which had been present for several months. He came to our clinic because of lower abdominal pain that started one month ago. He reported black stools, which were assessed as normal. He had been receiving medication for hypertension and atrial fibrillation. The tumor markers carcinoembryonic antigen (CEA) and carbohydrate antigen 19-9 (CA19-9) were elevated, measuring 40.7 (normal range: 0-0.5 ng/mL) and 70.4 (normal range: 0-37 U/mL), respectively. Esophagogastroduodenoscopy revealed an elevated lesion with ulceration at the esophagogastric junction (40 mm diameter, type 1 gross type) (Figures 1A, 1B), and biopsy revealed a well-differentiated adenocarcinoma. The human epidermal growth factor receptor 2 (HER2) score was immunohistochemistry (IHC)/2+ using the HercepTest. Chest and abdominal computed tomography (CT) showed wall thickening at the hilum and multiple enlarged lymph nodes in the left supraclavicular fossa and around the aorta from the hilum to the upper abdomen (Figures 2A, 2B, 2C). Positron emission tomography (PET)-CT confirmed wall thickening at the hilum with a maximum standardized uptake value (SUVmax) of 3.0 and multiple enlarged lymph nodes around the aorta from the hilum to the upper abdomen with an SUVmax ranging from 2.5 to 3.0 (Figures 3A, 3B). These findings were consistent with a malignant tumor at the esophagogastric junction, and esophagogastric junction cancer was diagnosed (T3N3M1 (LYM): stage IV).

**Figure 4:**
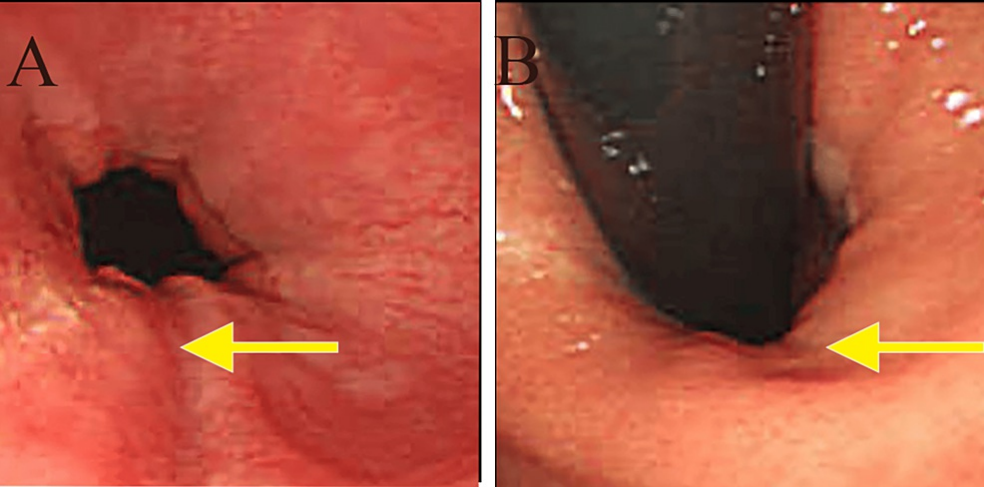
Esophagogastroduodenoscopy (A, B) Images taken after chemotherapy. Upper gastrointestinal endoscopy revealed that the tumor had almost disappeared.

**Figure 5:**
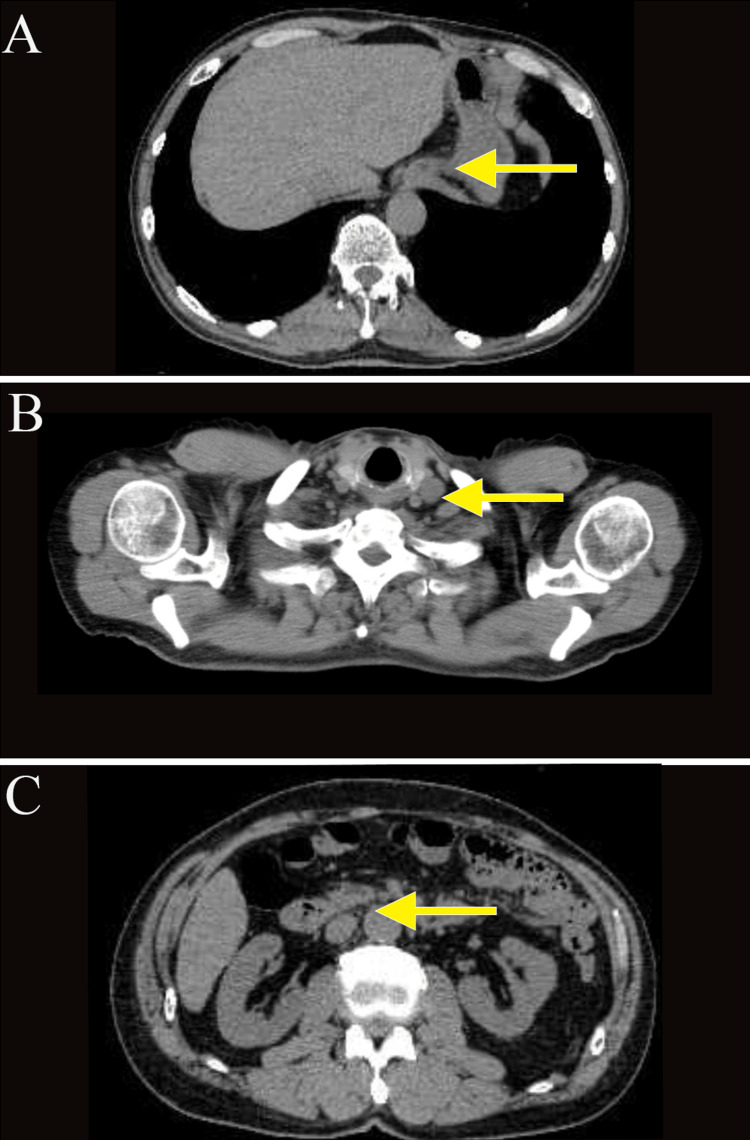
Computed tomography (A-C) Images taken after chemotherapy. CT scans showed that the tumor and multiple enlarged lymph nodes had almost disappeared.

**Figure 6:**
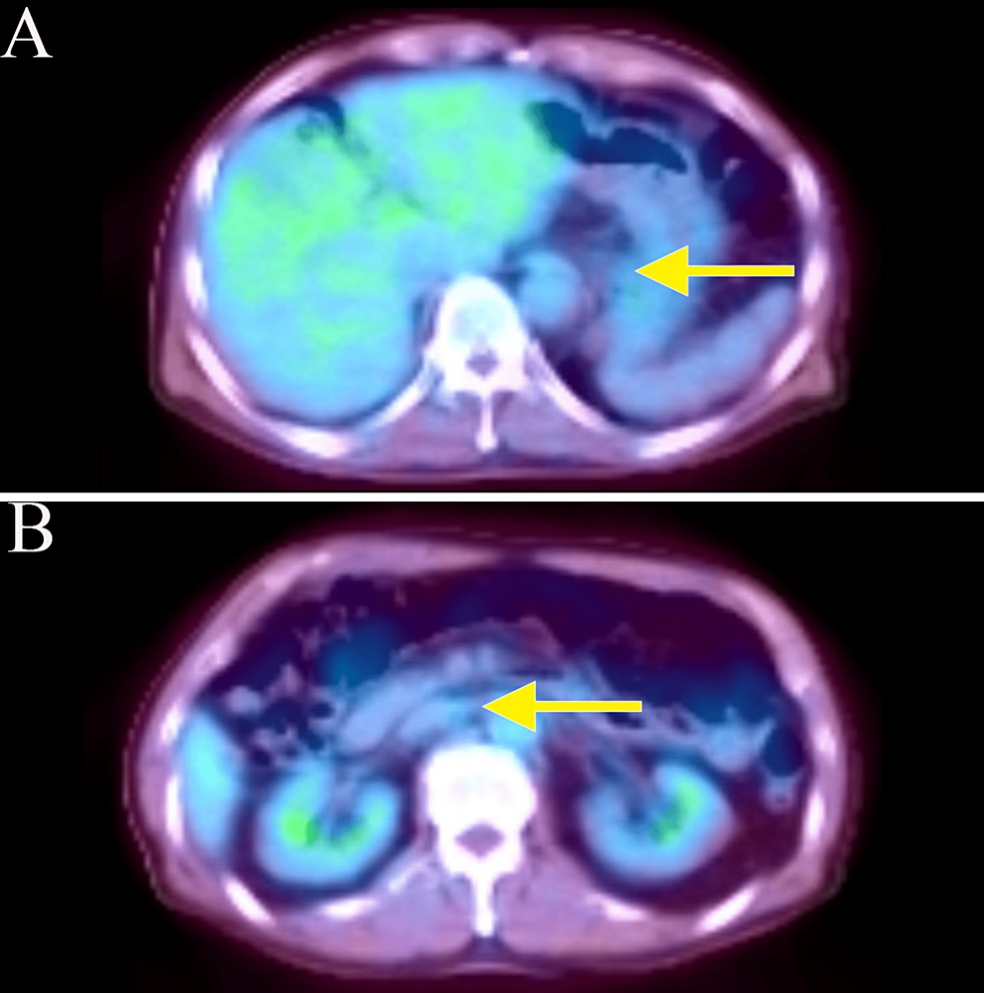
Positron emission tomography-computed tomography (A, B) Images taken after chemotherapy. PET-CT scans showed that the tumor had almost disappeared.

Chemotherapy was chosen and seven courses of S1 + cisplatin (SP) + trastuzumab (HCN) were administered; however, he developed chemotherapy-induced peripheral neuropathy, and SP was discontinued from the eighth course. Two courses of S1 + HCN were administered. Approximately 10 months after the start of chemotherapy, upper gastrointestinal endoscopy (Figures 4A, 4B), CT (Figures 5A, 5B, 5C), and PET-CT (Figures 6A, 6B) showed that the tumor had almost disappeared, and we decided to perform conversion surgery.

**Figure 7:**
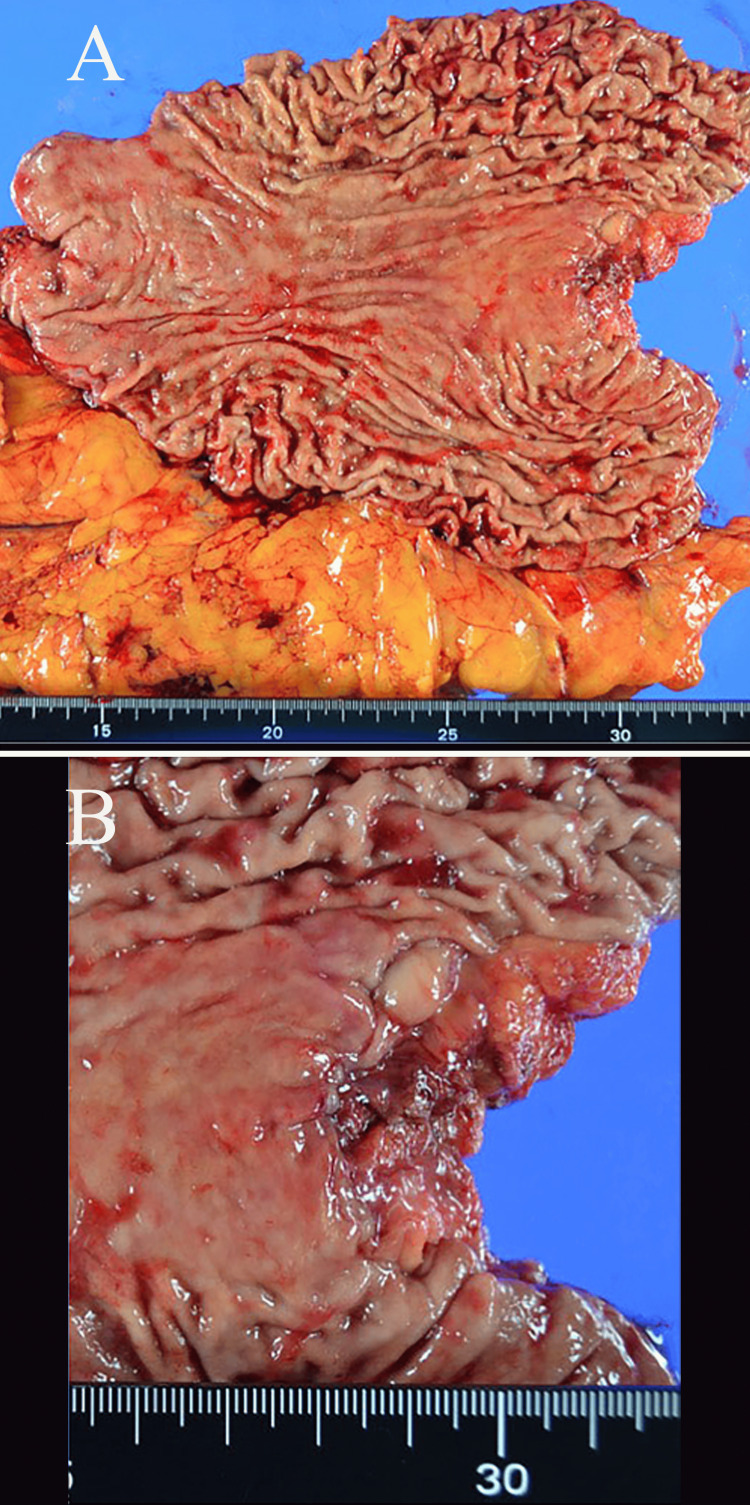
Resected specimen Scarring at the esophageal junction was evident upon palpation of the specimen.

**Figure 8:**
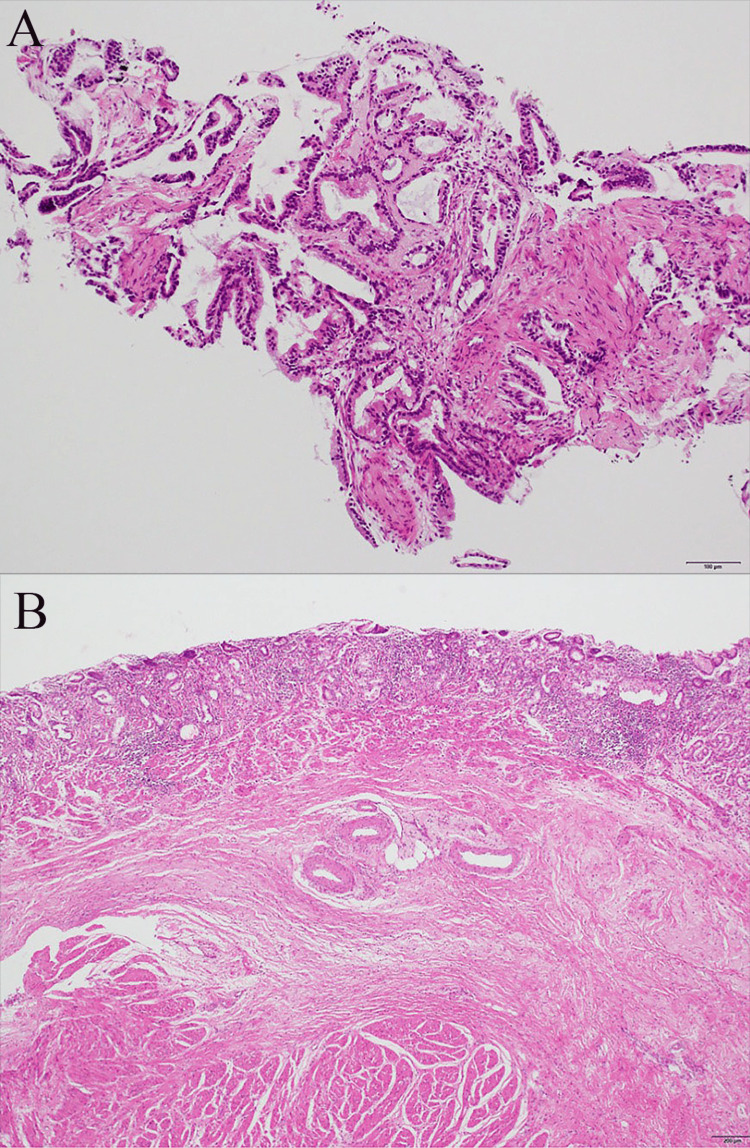
Pathological findings (hematoxylin and eosin stain) (A) The biopsy showed papillary adenocarcinoma and well-differentiated tubular adenocarcinoma. (B) Specimen showing scar tissue. Pathologic examination of the specimen and dissected lymph nodes indicated no cancer presence. The histological response grade of the treatment is grade 3.

At the beginning of surgery, we confirmed that the tumor was Cy0 (class I), P0, and the patient underwent open total gastrectomy, D2 lymph node dissection (Nos. 1, 2, 3, 4sa, 4sb, 4d, 5, 6, 7, 8, 9, and 11p; n=18), and Roux-en-Y reconstruction. Scarring at the esophageal junction was evident on palpation of the specimen, but pathologic examination of the specimen and dissected lymph nodes showed no cancer (Figures 7A, 7B and Figures 8A, 8B).

**Figure 9:**
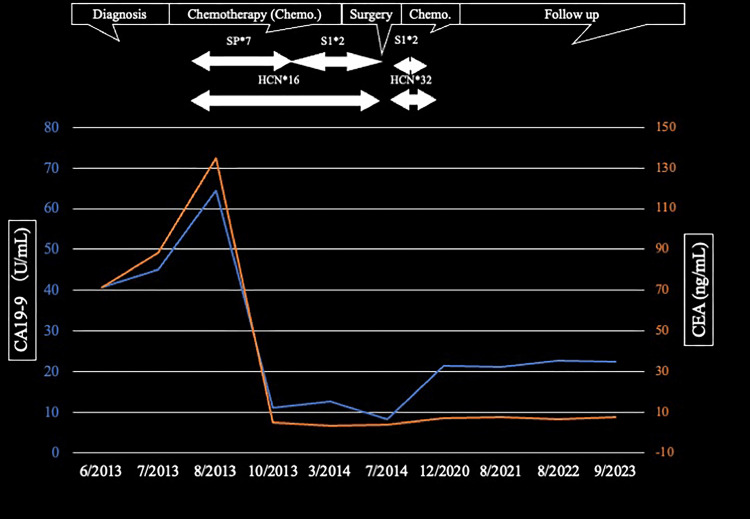
Time course of chemotherapy Chemotherapy was initiated with seven courses of S1 + cisplatin (SP) + trastuzumab (HCN). Starting with the eighth course, SP was discontinued. Two courses of S1 + HCN were administered postoperatively, followed by two cycles of S1 + HCN, and then 48 cycles of HCN alone until the second postoperative year. Nine years post-surgery, there were no signs of metastasis or recurrence, and the tumor markers were almost within normal limits. SP: S1 + cisplatin; HCN: trastuzumab; CEA: carcinoembryonic antigen; CA19-9: carbohydrate antigen 19-9

Postoperatively, the patient underwent two cycles of S1 + HCN followed by 48 cycles of HCN until the second postoperative year. There were no signs of metastasis or recurrence nine years after surgery, and the tumor markers were almost within normal limits (Figure 9).

## Discussion

Recent advances have improved response rates in patients with GC, but a complete cure remains challenging. Chemotherapy has evolved over the past decade, and several options are now available. The current patient was diagnosed with stage IV GC with enlarged left supraclavicular fossa and para-aortic lymph nodes and was treated with chemotherapy in our oncology department. Ten months later, no lesions were detected by endoscopy, CT, and PET-CT, and the patient felt that the chemotherapy had been effective. Following extensive discussion about possible surgery, we recommended conversion surgery and the patient agreed. The resected specimen showed no cancer in the stomach or lymph nodes. Postoperative chemotherapy was continued but was terminated midway. The patient had undergone 10 years of follow-up since the start of chemotherapy at the time of writing.

Although a complete response was confirmed by imaging and endoscopy in this case, surgery was deemed necessary [2], resulting in long-term recurrence-free survival. Kinoshita et al. [3] and Kano et al. [4] also recently reported on the use of conversion surgery for stage IV GC. Notably, we reviewed our case as category 2 with marginally resectable metastasis. According to the Japanese Gastric Cancer Treatment Guidelines 2018 (fifth edition), our treatment approach was aligned with recommended regimens. At that time, the regimen for postoperative care following conversion surgery with pathological complete response (pCR) was not well-defined. We considered using S1 with trastuzumab as a postoperative treatment for advanced cancer but ultimately discontinued S1 given the circumstances [5]. A previous report indicated that two courses of chemotherapy were acceptable, although four to six courses are usually administered, making the timing of chemotherapy before surgery challenging to determine [6].

Regarding postoperative chemotherapy, S1 alone is the preferred agent for esophagogastric junction cancer. In this case, however, the left supraclavicular and para-aortic lymph nodes were not dissected and preoperative S1 + HCN was considered. Notably, Yoshida et al. reported that chemotherapy should be continued for as long as possible after removal of the tumor, until the development of chemoresistance or uncontrollable adverse events [3]. The postoperative assessment revealed no signs of recurrence, and treatment continued for approximately two years, albeit with a brief discontinuation. Should a recurrence be identified, chemotherapy would be reconsidered. Genomic analysis revealed a subset of patients who responded well to chemotherapy, demonstrating its effectiveness [7]. The usefulness of HCN for HER2-positive GC is well known [8], and its use for GC expressing programmed death ligand 1 [9] and claudin 18.2 [10] has also recently been reported, and various clinical studies are underway. This report was based on a single institution’s experience, and further studies are needed to help determine the course of treatment in patients with GC.

The current patient initially wondered if he could be treated with anticancer drugs alone; however, he was unhappy about the prospects if the anticancer drugs stopped working. After the surgery, the patient reported feeling liberated, and was grateful for being able to lead a normal life for about 10 years.

## Conclusions

We report here the case of a patient with stage IV esophagogastric junction cancer who achieved a pathological complete response after SP + HCN therapy. Conversion surgery after chemotherapy resulted in recurrence-free survival. This case suggests that chemotherapy followed by R0 resection may lead to long-term survival in patients with HER2-positive stage IV esophagogastric junction cancer; however, further studies are needed to clarify the details of surgery after chemotherapy for stage IV GC, in line with the continuing evolution of chemotherapy.
